# Compression at Myofascial Trigger Point on Chronic Neck Pain Provides Pain Relief through the Prefrontal Cortex and Autonomic Nervous System: A Pilot Study

**DOI:** 10.3389/fnins.2017.00186

**Published:** 2017-04-11

**Authors:** Yoshiki Morikawa, Kouich Takamoto, Hiroshi Nishimaru, Toru Taguchi, Susumu Urakawa, Shigekazu Sakai, Taketoshi Ono, Hisao Nishijo

**Affiliations:** ^1^System Emotional Science, Graduate School of Medicine and Pharmaceutical Sciences, University of ToyamaToyama, Japan; ^2^Department of Judo Neurophysiotherapy, Graduate School of Medicine and Pharmaceutical Sciences, University of ToyamaToyama, Japan

**Keywords:** myofascial pain syndrome, massage, autonomic nervous system, pain, prefrontal cortex, near-infrared spectroscopy

## Abstract

Compression at myofascial trigger points (MTrPs), known as “ischemic compression,” has been reported to provide immediate relief of musculoskeletal pain and reduce the sympathetic activity that exacerbates chronic pain. We conducted a pilot study to investigate the possible involvement of the prefrontal cortex in pain relief obtained by MTrP compression in the present study, and analyzed the relationships among prefrontal hemodynamic activity, activity of the autonomic nervous system, and subjective pain in patients with chronic neck pain, with and without MTrP compression. Twenty-one female subjects with chronic neck pain were randomly assigned to two groups: MTrP compression (*n* = 11) or Non-MTrP compression (*n* = 10). Compression for 30 s was conducted 4 times. During the experiment, prefrontal hemodynamic activity [changes in Oxy-hemoglobin (Hb), Deoxy-Hb, and Total-Hb concentrations] and autonomic activity based on heart rate variability (HRV) were monitored by using near infrared spectroscopy (NIRS) and electrocardiography (ECG), respectively. The results indicated that MTrP compression significantly reduced subjective pain compared with Non-MTrP compression. The spectral frequency-domain analyses of HRV indicated that a low frequency (LF) component of HRV was decreased, and a high frequency (HF) component of HRV was increased during MTrP compression, while LF/HF ratio was decreased during MTrP compression. In addition, prefrontal hemodynamic activity was significantly decreased during MTrP compression compared with Non-MTrP compression. Furthermore, changes in autonomic activity were significantly correlated with changes in subjective pain and prefrontal hemodynamic activity. Along with previous studies indicating a role for sympathetic activity in the exacerbation of chronic pain, the present results suggest that MTrP compression in the neck region alters the activity of the autonomic nervous system via the prefrontal cortex to reduce subjective pain.

## Introduction

The prevalence of neck pain in the general population has been increasing in recent years (Hoy et al., 2010), and women complain more frequently of neck pain than men (Guez et al., 2002; Côté et al., 2004; Fejer et al., 2006). Musculoskeletal neck pain is reportedly caused by myofascial trigger points (MTrPs) in the neck and shoulder muscles (Simons et al., 1999). MTrPs are hypersensitive spots in palpable taut bands of skeletal muscle fibers (Simons et al., 1999), and recent clinical studies have reported that patients with chronic neck pain have a larger number of MTrPs in the upper trapezius muscle than healthy subjects (Fernández-de-las-Peñas et al., 2007; Muñoz-Muñoz et al., 2012). These studies suggest that MTrPs are responsible for chronic neck pain.

Abnormalities of the autonomic nervous system are thought to be involved in the maintenance of chronic musculoskeletal pain (Passatore and Roatta, 2006; Martinez-Lavin, 2007). Excessive activity of the sympathetic nervous system and reduced activity of the parasympathetic nervous system have been reported in patients with chronic pain such as neck-shoulder pain and fibromyalgia (Gockel et al., 1995; Furlan et al., 2005; Hallman et al., 2011). Altered sympathetic activity with sweating, vasoconstriction, vasodilation, and piloerection has also been reported in MTrP regions (Simons et al., 1999). Increased sympathetic activity is thought to exacerbate spontaneous local pain at MTrPs in patients with chronic neck and shoulder pain (Ge et al., 2006). Thus, abnormalities of the autonomic nervous system seem to be involved in the chronic pain associated with MTrPs.

Recent human neuroimaging studies have shown that several brain regions including the prefrontal cortex are involved in the processing of pain information as well as autonomic regulation (Hallman and Lyskov, 2012). In those with chronic pain, anatomical and functional abnormalities have been found in the medial prefrontal cortex (mPFC) and higher ratings of spontaneous pain were associated with increased activity in the mPFC of patients with chronic pain (Baliki et al., 2006). Furthermore, a study using functional magnetic resonance imaging (fMRI) has suggested that the mPFC is involved in the generation of autonomic responses (Critchley et al., 2000). These results imply that the prefrontal cortex is involved in chronic pain through abnormal activation of the autonomic nervous system.

Compression at MTrPs is an effective massage technique for musculoskeletal pain, and can provide immediate relief from chronic neck pain (Hou et al., 2002), with increased parasympathetic activity and decreased sympathetic activity (Delaney et al., 2002; Takamoto et al., 2009). MTrP compression is considered to exert its therapeutic effects through peripheral, spinal, supraspinal, and autonomic pathways (Bialosky et al., 2009; Takamoto et al., 2009). However, its effects on pain perception and autonomic control in the prefrontal cortex remain unclear.

In the present study, we hypothesized that MTrP compression might affect the mPFC involved in autonomic regulation, which in turn induces pain relief. To investigate possible involvement of the mPFC in pain relief by MTrP compression, we analyzed the relationships among prefrontal hemodynamic activity measured using near infrared spectroscopy (NIRS), activity of the autonomic nervous system based on heart rate variability (HRV), and subjective pain in patients with chronic neck pain, with and without treatment by MTrP compression. NIRS is a non-invasive neuroimaging technique that can measure changes in oxy-hemoglobin (Oxy-Hb), deoxy-hemoglobin (Deoxy-Hb), and total hemoglobin (Total-Hb) in the cortical surface elicited by local cortical neuronal activities (Ferrari and Quaresima, 2012). It is noted that NIRS can be applied with less body and head restriction in a limited space, compared with the other non-invasive methods including fMRI and positron emission tomography (PET). Thus, NIRS allows to measure brain activity in a similar condition to that in actual clinical environments.

## Materials and methods

### Subjects

**Figure 1:**
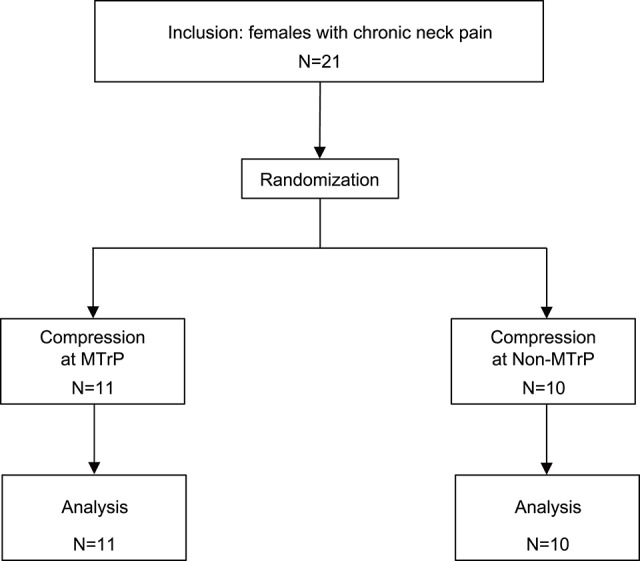
**Participant flow diagram**.

The participants comprised 21 female patients (aged 20–31 years; 23.4 ± 0.9 years, mean age ± standard error [SE]) who had complained of neck pain lasting more than 3 months from the time of onset. All patients were diagnosed with myofascial-pain syndrome by a well-trained clinical practitioner with a national license to practice as an acupuncturist. Inclusion criteria were (1) a palpable band or hardened nodules in the upper trapezius muscle, and (2) myofascial pain emanating from a well-localized area in the palpable band. These patients were randomly assigned to two groups (MTrP and Non-MTrP groups) (Figure 1), based on a random allocation software. All patients were treated in strict compliance with the Declaration of Helsinki and the U.S. code of Federal Regulations for the Protection of Human Subjects. The experiments were conducted with the understanding and informed written consent of each subject, and approved by the Clinical Research and Ethics Committee at the University of Toyama.

### Muscle compression

Ischemic compression consisted of constant deep pressure by the thumb, which was applied to MTrPs or Non-MTrPs in the trapezius muscle. The locations of the MTrPs in the trapezius muscle were identified by a practitioner with a national license to practice acupuncture in Japan who had more than 6 years' experience in MTrPs diagnosis and treatment. The identification of MTrPs was based on the minimal diagnostic criteria recommended by Simons et al. (1999) and Gerwin et al. (1997), requiring (1) the presence of a hypersensitive tender point in a taut band in the muscle, (2) recognition by the patient of the pain as “familiar” when compression is applied to the point, and (3) induction of pain when the muscle including the MTrP is stretched. For the controls, Non-MTrPs were defined as points 2 cm away and proximal to a given MTrP, where a taut band was not detected and local and referred pain was not induced when compression was applied to the point.

The intensity of compression was set at a point midway between the pressure pain threshold (PPT) and maximally tolerable pain (MTP), which corresponded to a “moderately painful average” intensity in each patient (Hou et al., 2002). PPTs and MTPs were measured using a digital algometer (Atkins et al., 1992; Bendtsen et al., 1994). The device consisted of a capacitance pressure sensor with a circular tip (diameter, 6 mm) (HV-2-556: SHOWA SOKKI, Japan) and data collection hardware connected to the sensor (SME-101A: Kyowa Dengyou, Japan). The sensor was attached to the pad of the thumb. Pressure intensity was expressed in Arbitrary Unit (AU); 550 AU is equivalent to about 1 kg. Pressure intensity was displayed on a device monitor that was not visible to the palpating practitioner or the patients. Two experimenters worked together during data collection. Experimenter 1 (palpating practitioner), who was blinded to the monitor recordings, determined the PPT and MTP based on the behavioral responses of the patient, while Experimenter 2 recorded the pressure intensity values from the device monitor. Experimenter 1 applied steady, gradually increasing pressure to the identified MTrPs or Non-MTrPs using the thumb with the pressure sensor. The patients were instructed to press a button when they initially felt pain and also when they felt severe pain, where 0 = no pain, 4 = pain threshold (PPT), and 10 = severe pain. Compression ceased when patients felt severe pain, and Experimenter 2 recorded the pressure intensity values. The PPT and MTP were recorded 3 times for each stimulation site, and the intensity of compression in the later experiment was set at an intensity midway between the mean PPT and mean MTP for each subject.

### Protocol

**Figure 2:**
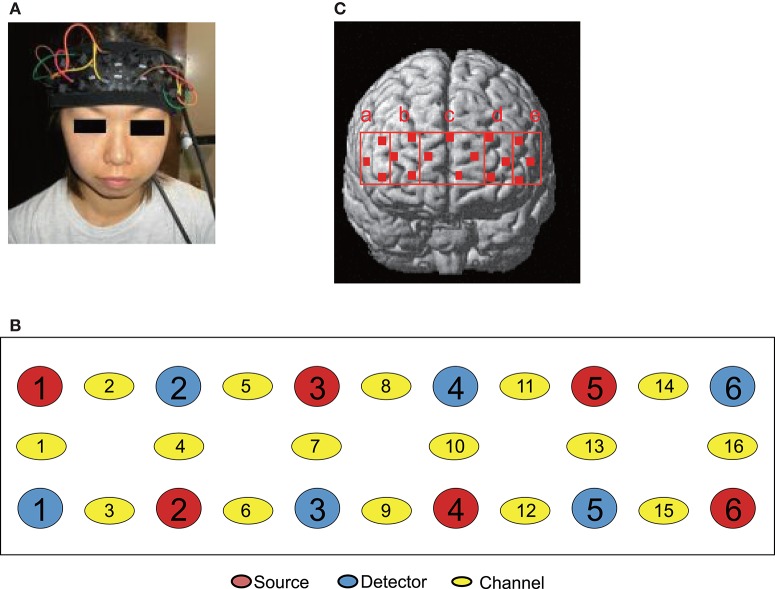
**Location of NIRS probes and channels. (A)** Location of the NIRS head pad on the frontal region. **(B)** The arrangement of probes (sources and detectors) and channels. **(C)** Spatial estimation of NIRS channels. The coordinates of each NIRS channel were normalized to MNI (Montreal Neurological Institute) space using virtual registration. The red dots indicate the coordinates of the channels: a, right dorsolateral prefrontal cortex (rDLPFC) (Brodmann Areas 45, 46); b, right dorsomedial prefrontal cortex (rDMPFC) (Brodmann Area 10); c, central dorsomedial prefrontal cortex (cDMPFC) (Area 10); d, left dorsomedial prefrontal cortex (lDMPFC) (Area 10); e, left dorsolateral prefrontal cortex (lDLPFC) (Areas 45, 46).

The patients were fitted with a NIRS head pad on the frontal region (Figure 2A) and ECG electrodes on the chest. The patients lay in a supine position on a bed with the cervical spine in a neutral position. The patients were instructed to close their eyes and relax as much as possible. Muscle compression was started 1 min after the commencement of NIRS and ECG recordings. Compression was applied to MTrPs or Non-MTrPs for 30 s, followed by a rest for 120 s. This cycle was repeated 4 times (4 cycles). The practitioner maintained constant pressure (i.e., a pressure intensity midway between the PPT and MTP) by monitoring the pressure intensity of the thumb.

### Psychophysical data

To subjectively evaluate the compression treatment, the subjects were instructed to report pain levels in the neck before and after the experiment using a 100-grade visual analog scale (VAS) that is measured in mm. In subjective pain scoring, score 0 indicted “no pain,” and score 100, “strongest pain perception that the subjects have ever had.” At the end of the experiment, the subjects were also asked to report the pleasantness of the compression in each cycle on a scale of –10 to 10, where the extremes represented the most unpleasant and pleasant experiences, respectively (the comfort/discomfort score). The comfort/discomfort scores from 4 cycles were averaged. Furthermore, the subjects reported the pain intensity during compression in each cycle using a 10-grade scale, where a score of 0 indicated “no pain,” a score of 4 indicated “pain sensation at a threshold level,” and score 10 indicated “strongest pain perception” (pain intensity score). The pain intensity scores from 4 cycles were averaged.

### Measurement of the activity of the autonomic nervous system

Autonomic activity was measured using a system for measuring HRV (Makin AD2; Suwa Trust, Japan). The V5 chest leads of the ECG were used. RR (beat-to-beat) intervals were analyzed by the maximum-entropy method to calculate the HRV spectrum. The low-frequency (LF) (0.04–0.15 Hz) and high-frequency (HF) (0.15–0.40 Hz) components of heart variability (HRV) were calculated. In addition, the LF/HF ratio was obtained. The LF/HF ratio has been used as an index of a sympatho-vagal balance, the LF component as an index of sympathetic modulation, and the HF component as an index of parasympathetic (vagal) modulation (Task Force of the European Society of Cardiology and the North American Society of Pacing and Electrophysiology, 1996; Pagani et al., 1997; Thayer et al., 2010). However, recent studies indicate that meanings of these indices are more complex (see Limitations) (Billman, 2011, 2013). Furthermore, these indices are also modulated by several non-autonomic nervous factors such as respiration and heart rate themselves (Billman, 2013; Gasior et al., 2016). Therefore, respiratory rates were estimated from the R-R intervals of ECG data using a free software (Kubios HRV ver.2.2; http://www.kubios.com; University of Eastern Finland, Kuopio, Finland; Tarvainen et al., 2014).

### Measurement of cerebral hemodynamic activity in the prefrontal cortex

A NIRS instrument (Spectratech OEG-16; Spectratech Inc., Yokohama, Japan) was used to measure cerebral hemodynamic activity. Six optical sources and 6 detectors (resulting in a total of 16 recording channels) were fixed on the frontal area (Figures 2A,B). The bottom horizontal line of the probes was placed on the Fp1–Fp2 line in the 10–20 EEG system. The sources and detectors were positioned so that distance between any 2 probes was set at 3 cm. The midpoints between a source and a detector were called “channels,” and hemodynamic activity was detected by pairs of source and detector using two different wavelengths (770 and 840 nm). Changes in the hemoglobin (Hb) concentration (ΔOxy-Hb, ΔDeoxy-Hb, and ΔTotal-Hb [ΔOxy-Hb + ΔDeoxy-Hb]) from the baseline were estimated using a modified Lambert-Beer law (Seiyama et al., 1988; Wray et al., 1988). After the recording, the 3-D locations of the NIRS probes were measured using a digitizer (Shimadzu Co. Ltd., Japan) with reference to the nasion and bilateral external auditory meatus.

### Data analysis

The data are presented as the mean ± standard error (SE). Normality of the data distribution was assessed by Shapiro-Wilk test. The homogeneity of variance in all variables with normality was assessed by the Levene's test. Student's *t*-test (or Mann-Whitney U test) and ANOVA were used to compare the data in analyzed parameters between the MTrP and Non-MTrP groups. When the data during compression were compared between the MTrP and Non-MTrP groups, the data (VAS, heart rate, respiratory rate, and HRV parameters) during compression were corrected for the baselines.

The HF and LF components were reported as percentages (HF%, LF%, respectively), and normalized by dividing them by the sum of all components (LF + HF). This normalization of LF and HF is recommended since it tends to minimize the effect on the values of LF and HF components of the changes in total power (Task Force of the European Society of Cardiology and the North American Society of Pacing and Electrophysiology, 1996; Pagani et al., 1997). Furthermore, the low values of HF, LF, and LF/HF were also normalized by natural logarithm transformation (lnLF, lnHF, lnLF/HF, respectively). Changes in HRV after the treatments were assessed by subtracting the mean value of each parameter in the 30-s rest period before the application of compression from the mean value for the 30-s period during compression. All autonomic data (changes in HR, respiratory rate, and HRV parameters) were averaged across 4 cycles, since the all autonomic parameters except respiratory rate showed no significant main effect of “cycle” (*P* > 0.05) by repeated measures one-way ANOVAs with a factor of “cycle” in each treatment group while respiratory data showed no significant effect of “cycle” (*P* > 0.05) by Friedman's test. Student's *t*-test was used to compare changes in autonomic activity between the MTrP and Non-MTrP groups. Findings of *P* < 0.05 were considered significant.

**Figure 3:**
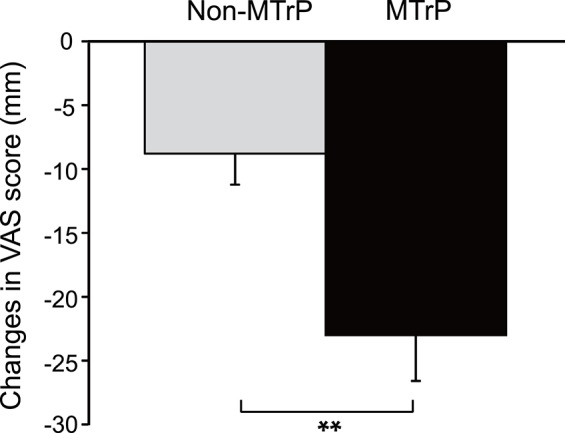
**Comparison of changes in subjective pain scores after compression between the MTrPs and Non-MTrP groups**. Ischemic compression at MTrPs significantly decreased subjective pain scores compared with Non-MTrP compression. Error bars indicate SEM. VAS, visual analog scale; ^**^P < 0.01.

To identity the anatomical locations of NIRS channels in each subject, the 3D locations of the NIRS probes and channels in each subject were spatially normalized to a standard coordinate system using NIRS SPM software (statistical parametric mapping: http://bisp.kaist.ac.kr/NIRS-SPM, version 3.1; Ye et al., 2009); the coordinates for each NIRS channel were normalized to MNI (Montreal Neurological Institute) space using virtual registration (Tsuzuki et al., 2007). We then identified the Brodmann areas corresponding to the NIRS channels of each subject using MRIcro software (www.mricro.com, version 1.4). We divided the channels into 5 regions: the dorsolateral prefrontal cortex (DLPFC; Brodmann areas 45 and 46) in each hemisphere corresponding to Ca (rDLPFC) and Ce (lDLPFC) in Figures 2, 3 regions in the dorsomedial prefrontal cortex (DMPFC; Brodmann area 10) corresponding to Cb (rDMPFC), Cc (cDMPFC), and Cd (lDMPFC) in Figure 2. In two subjects, the locations of NIRS channels were determined by stereotaxic superimposition on the surface of the 3-D MRI reconstructed brain of each subject. For 3-D MRI, thin-slice 3-D sagittal T1-weighted gradient echo MR images were obtained at 1.5 T using a specific protocol tailored for reconstruction (Takeuchi et al., 2009). These two subjects had the following protocol: (TR/TE/NSA) 25/5/1, flip angle 10, FOV 87.5 cm, matrix 256 9 256, 1.0 mm contiguous slices, obtained in a plane parallel to the brain stem.

Cerebral hemodynamic changes during compression at MTrPs and Non-MTrPs were converted to effect sizes. Effect sizes can adjust for the influence of different path-length factors among different subjects and cortical regions (Schroeter et al., 2003; Suzuki et al., 2008). The effect sizes of hemodynamic responses were calculated according to the following formula: effect size = [(mean Oxy-Hb levels during compression for 30 s) − (mean Oxy-Hb levels during the rest period for 30 s before start of compression)]/[standard deviation of Oxy-Hb levels during the rest period of 30 s before the start of compression]. For each channel, the effect sizes from 4 cycles were averaged. The data from the channels in each brain region were then averaged to give the mean hemodynamic responses in each area for each patient. These data for compression at MTrPs and Non-MTrPs were compared using a repeated-measures two-way ANOVA (treatment × brain region). Findings of *P* < 0.05 were considered significant.

We also analyzed correlations among the effect sizes of hemodynamic responses in the DMPFC, changes in possible autonomic activity with compression (HF%, LF%, LF/HF, lnHF, lnLF, and lnLF/HF), and changes in subjective pain scores with compression (VAS scores) using simple regression analysis.

All data analyses were performed using SPSS 19.0 (IBM Inc., New York, USA). A *p* < 0.05 was considered statistically significant.

### Sample size

Sample size for comparison of two independent samples (two-tailed *t*-test) was estimated using a free sample size calculator (https://www.stat.ubc.ca/~rollin/stats/ssize/n2.html by Dr. Rollin Brant, University of British Columbia) as *n* = 10 based on the following condition; changes in VAS in the MTrP group = −35, changes in VAS in the Non-MTrP group = −16, standard deviation (SD) = 15, level of significance = 0.05, statistical power = 0.8. Our previous preliminary data were used for this sample size estimation.

## Results

### Baseline characteristics and sensations evoked by compression in the two groups

Table 1 shows the baseline clinical characteristics of the patients (age, VAS score for neck pain, PPT, and intensity of compression, heart rate, and respiratory rate). There were no significant differences between the two groups in the baseline characteristics of these parameters (Student's *t*-test, *P* > 0.05). Furthermore, there were no significant differences in the sensations evoked by compression between the two groups (pain intensity scores, comfort/discomfort scores) (Student's *t*-test, *P* > 0.05).

**Table 1:** **Baseline characteristics and sensations evoked by compression in the two groups**.

	**Non-MTrP (*N* = 10)**	**MTrP (*N* = 11)**
Age (years)	23.0 ± 1.0	23.8 ± 0.9
Stimulation side (R/L)	6/4	7/4
VAS (mm)	55.4 ± 5.5	53.4 ± 5.2
PPT (AU)	618.5 ± 80.4	517.1 ± 76.6
Intensity of compression (AU)	789.7 ± 79.9	667.8 ± 76.2
Pain intensity score during compression	6.8 ± 0.3	6.6 ± 0.3
Comfort/discomfort score during compression	6.6 ± 0.5	5.6 ± 0.5
Heart rate (beats/min)	65.6 ± 2.5	59.5 ± 2.1
Respiration rate (Hz)	0.20 ± 0.005	0.21 ± 0.007

*Data are presented as mean ± SE. R/L, Right side/Left side; VAS, Visual Analog Scale; PPT, pressure pain threshold. There were no significant differences in these parameters between the MTrP and Non-MTrP groups (Student's t-test, P > 0.05)*.

### Changes in subjective pain scores in the neck

Figure 3 compares the changes in subjective pain scores resulting from compression in the MTrP and Non-MTrP groups. Compression at MTrPs significantly ameliorated subjective pain scores compared with compression at Non-MTrPs (Student's *t*-test, *P* < 0.01).

### Changes in heart rate variability from the pre-treatment baseline

**Figure 4:**
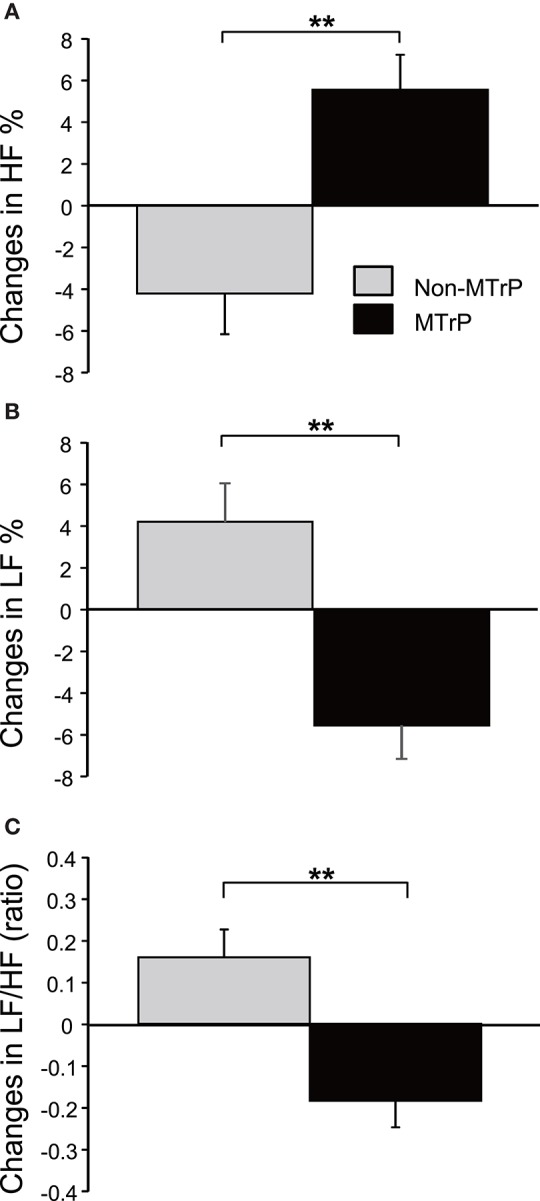
**Comparison of changes in the activity of the autonomic nervous system during ischemic compression between the MTrP and Non-MTrP groups. (A)** High frequency (HF) components of HR variability (HF%) were significantly increased during ischemic compression at MTrPs compared with Non-MTrPs. **(B)** Low frequency (LF) components of HR variability (LF%) was significantly decreased during ischemic compression at MTrPs compared with Non-MTrPs. **(C)** LF/HF ratio was significantly decreased during ischemic compression at MTrPs compared with Non-MTrPs. Error bars indicate SE; ^**^P < 0.01.

There was no significant difference in changes in heart rates during compression between the MTrP and Non-MTrP groups (Student's *t*-test, *P* > 0.05) (Supplementary Figure 1A), nor significant difference in changes in respiratory rates during compression between the MTrP and Non-MTrP groups (Mann-Whitney U test, *P* > 0.05) (Supplementary Figure 1B). Figure 4 compares changes in autonomic responses during compression between the MTrP and Non-MTrP groups. The HF components (HF%) were significantly greater in the MTrP group compared with the Non-MTrP group (Student's *t*-test, *P* < 0.01) (A), while the LF components (LF%) were significantly greater in the MTrP group compared with the Non-MTrP group (Student's *t*-test, *P* < 0.01) (B). The LF/HF ratio was significantly less in the MTrP group compared with the Non-MTrP group (Student's *t*-test, *P* < 0.01) (C). These results suggest that compression at MTrPs suppressed sympathetic activity.

We also analyzed logarithmically transformed HRV parameters (Supplementary Figures 1C–E). The trend of the results was essentially consistent with that in Figure 4. Changes in lnLF during compression were significantly less in the MTrP group compared with the Non-MTrP group (Student's *t*-test, *P* < 0.01) (C), while there were no significant differences in changes in lnHF during compression between the MTrPs and Non-MTrPs (Student's *t*-test, *P* > 0.05) (D). Furthermore, changes in lnLF/HF during compression were significantly less in the MTrP group compared with the Non-MTrP group (Student's *t*-test, *P* < 0.01) (E).

### Prefrontal hemodynamic responses

**Figure 5:**
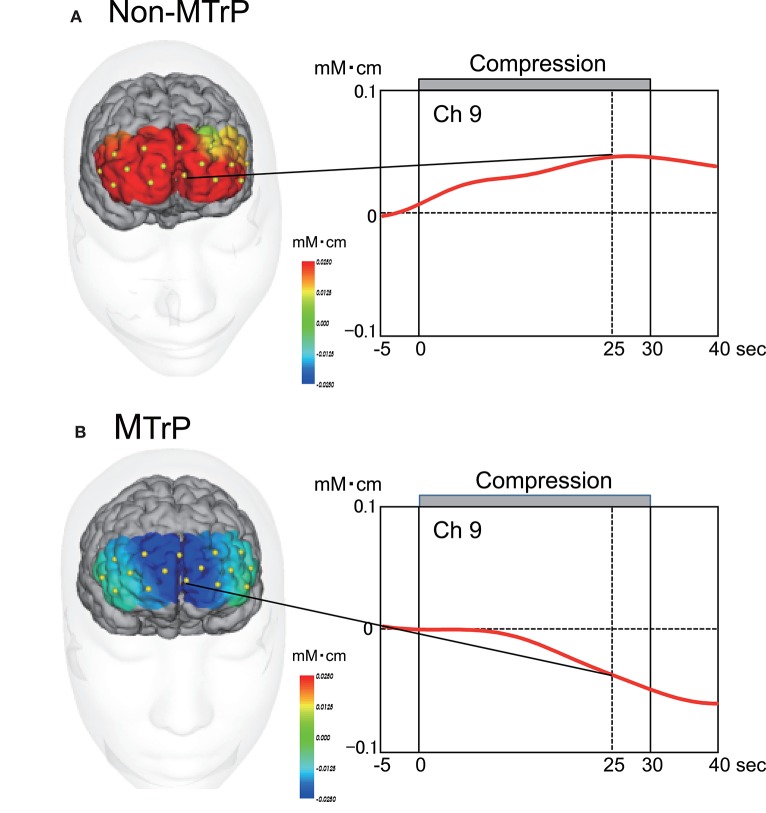
**Time course and topographical maps of Oxy-Hb changes during Non-MTrP (A)** and MTrP **(B)** compression. Topographical maps indicate Oxy-Hb maps 25 s after starting ischemic compression. NIRS image data were superimposed on individual 3D-MRIs. Yellow dots on the 3D-MRI indicate NIRS channels. Red lines indicate changes in Oxy-Hb concentrations from baseline activity. Note that the Oxy-Hb concentration in the prefrontal cortex was decreased during MTrP compression.

Figure 5 shows typical examples of Oxy-Hb maps 25 s after starting compression at Non-MTrPs (A) and MTrPs (B). The NIRS data were stereotaxically superimposed on the 3D-MRIs of the brain of the subject to construct the Oxy-Hb concentration maps. The Oxy-Hb concentration in the DMPFC gradually increased during compression at Non-MTrPs (A), while it decreased during compression at MTrPs (B).

**Figure 6:**
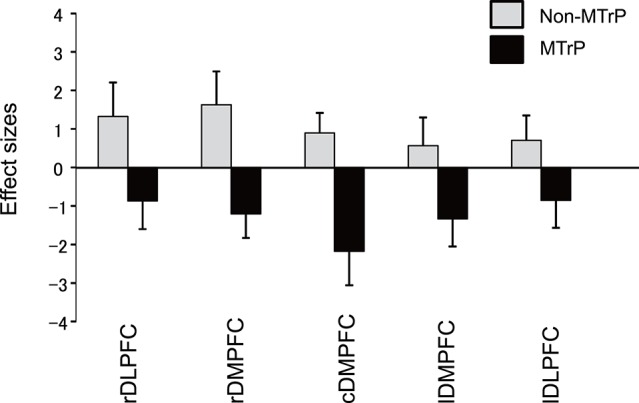
**Comparison of hemodynamic responses in the prefrontal regions during ischemic compression between MTrPs and Non-MTrP groups**. Hemodynamic responses are shown as effect sizes. Statistical analysis by repeated-measures 2-way ANOVA indicated that MTrP compression significantly decreased the effect size compared with Non-MTrP compression. Error bars indicate SE. rDLPFC, right dorsolateral prefrontal cortex; rDMPFC, right dorsomedial prefrontal cortex; cDMPFC, central dorsomedial prefrontal cortex; lDMPFC, left dorsomedial prefrontal cortex; lDLPFC, left dorsolateral prefrontal cortex.

Figure 6 compares the mean effect sizes of hemodynamic responses in the 5 prefrontal regions of the MTrP and Non-MTrP groups. Statistical analysis of the data using a repeated-measures 2-way ANOVA with “treatment” (MTrP vs. Non-MTrP) and “brain region” as factors indicated that there was a significant main effect of treatment [*F*_(1, 19)_ = 6.624, *P* < 0.05], but not brain region [*F*_(2.697, 51.236)_ = 1.672, *P* > 0.05], and no significant interaction between treatment and brain region [*F*_(2.697, 51.236)_ = 1.179, *P* > 0.05]. The results indicate that compression at MTrPs significantly decreased hemodynamic activity in the prefrontal cortex. In contrast, there was no significant differences in Deoxy-Hb between the MTrP and Non-MTrP group in the same statistical analysis using a repeated measures 2-way ANOVA; there was no significant main effect of treatment [*F*_(1, 19)_ = 0.019, *P* > 0.05], nor significant interaction between treatment and brain region [*F*_(2.8, 53.73)_ = 0.664, *P* > 0.05].

### Relationships among subjective pain, autonomic activity, and prefrontal hemodynamic responses

**Figure 7:**
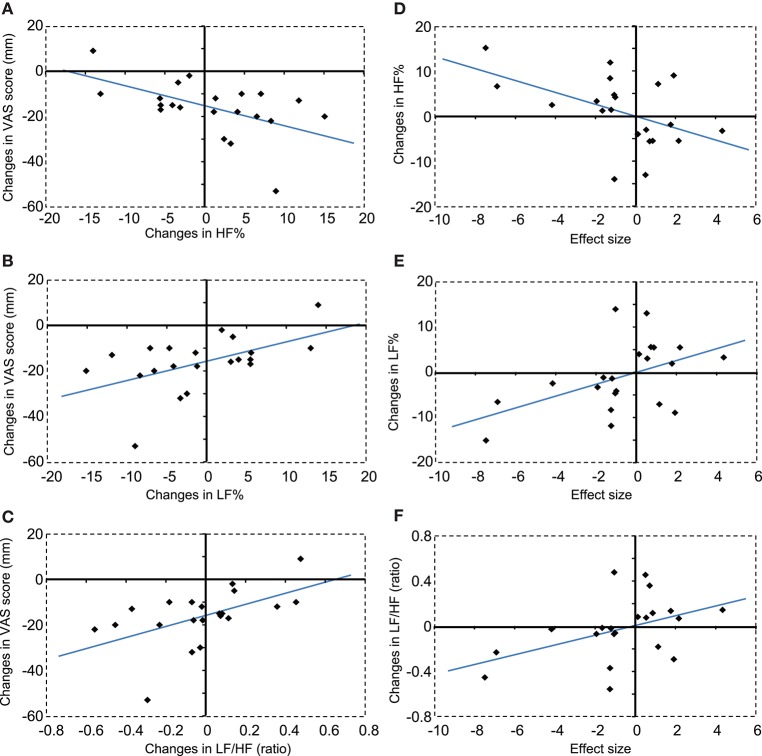
**Correlations between changes in autonomic activity and changes in subjective pain scores (A–C)**, and between changes in autonomic activity and changes in hemodynamic responses in the DMPFC **(D–F)**. **(A)** A negative correlation was observed between changes in HF% and changes in subjective pain scores. VAS, visual analog scale. **(B)** A positive correlation was observed between changes in LF% and changes in subjective pain scores. **(C)** A positive correlation was observed between changes in LF/HF ratios and changes in subjective pain scores. **(D)** A negative correlation was observed between changes in HF% and changes in the hemodynamic response in the DMPFC. **(E)** A positive correlation was observed between changes in LF% and changes in the hemodynamic response in the DMPFC. **(F)** A positive correlation was observed between changes in LF/HF ratios and changes in the hemodynamic response in the DMPFC.

Figures 7A–C show the correlations between changes in autonomic activity with compression and changes in subjective pain scores. Changes in HF% were significantly and negatively correlated with changes in subjective pain scores, *r*^2^ = 0.272, *F*_(1, 20)_ = 7.092, *P* < 0.05 (A). In contrast, changes in LF% were significantly and positively correlated with changes in subjective pain scores, *r*^2^ = 0.272, *F*_(1, 20)_ = 7.092, *P* < 0.05 (B). Furthermore, changes in LF/HF were significantly and positively correlated with changes in subjective pain scores, *r*^2^ = 0.285, *F*_(1, 20)_ = 7.573, *P* < 0.05 (C).

Figures 7D–F show correlations between changes in autonomic activity and the mean effect sizes of cerebral hemodynamic responses in the cDMPFC during compression. Changes in HF% were significantly and negatively correlated with the effect sizes of hemodynamic responses in the DMPFC, *r*^2^ = 0.235, *F*_(1, 20)_ = 5.830, *P* < 0.05 (D). In contrast, changes in the LF% were significantly and positively correlated with the effect size of hemodynamic responses in the DMPFC, *r*^2^ = 0.235, *F*_(1, 20)_ = 5.830, *P* < 0.05 (E). Furthermore, changes in the LF/HF ratio were significantly and positively correlated with the effect size of hemodynamic responses in the DMPFC, *r*^2^ = 0.192, *F*_(1, 20)_ = 4.514, *P* < 0.05 (F). However, there was no significant correlation between changes in subjective pain scores and the effect size of hemodynamic responses in the DMPFC (data not shown).

We also analyzed logarithmically transformed parameters (lnLF, lnHF, and lnLF/HF) (Supplementary Figure 2). The results were essentially consistent with those in Figure 7. Changes in lnLF were significantly and positively correlated with changes in subjective pain scores, *r*^2^ = 0.299, *F*_(1, 20)_ = 8.121, *P* < 0.05 (A). Furthermore, changes in lnLF/HF were significantly and positively correlated with changes in subjective pain scores, *r*^2^ = 0.332, *F*_(1, 20)_ = 9.439, *P* < 0.05 (B). However, there was no significant correlation between changes in lnHF and changes in subjective pain scores (data not shown). On the other hand, changes in lnHF were significantly and negatively correlated with the effect size of hemodynamic responses in the DMPFC, *r*^2^ = 0.205, *F*_(1, 20)_ = 4.890, *P* < 0.05 (C). Furthermore, changes in lnLF/HF were significantly and positively correlated with the effect size of hemodynamic responses in the DMPFC, *r*^2^ = 0.247, *F*_(1, 20)_ = 6.223, *P* < 0.05 (D). However, there was no significant correlation between changes in lnLF and the effect size of hemodynamic responses in the DMPFC (data not shown).

## Discussion

### Effects of MTrP compression on pain perception and the activity of the autonomic nervous system

In the present study, compression at MTrPs significantly ameliorated subjective pain compared with compression at Non-MTrPs (Figure 3). It also significantly increased HRV parameters, which are believed to reflect parasympathetic activity, and inhibited HRV parameters, which are believed to reflect sympathetic activity (Figure 4, Supplementary Figure 1). Furthermore, these changes in autonomic activity were significantly correlated with changes in subjective pain scores (Figure 7, Supplementary Figure 2). Consistent with the present study, previous physiological studies have reported that compression at MTrPs increases the activity of the parasympathetic nervous system and decreases sympathetic activity (Delaney et al., 2002; Takamoto et al., 2009). Furthermore, a reduction of sympathetic activity during thermotherapy for the neck has been associated with decreased neck stiffness and fatigue (Yasui et al., 2010). These findings suggest that altered sympathetic activity might exacerbate neck pain, and that compression at MTrPs might reduce neck pain by suppressing sympathetic activity.

Acute or chronic muscle overload induces hyperactivation of the neuromuscular junction, which leads to an excessive release of acetylcholine (Gerwin et al., 2004; Bron and Dommerholt, 2012). This excess of acetylcholine at the motor endplates leads to the formation of knots in muscle fibers that are characterized by continuous contraction. Formation of contraction knots then leads to the development of local ischemia and hypoxia. The loss of energy and O_2_ supply in contracting muscle causes the release of sensitizing noxious substances that lead to increased hypersensitivity and pain. Increased activity of the sympathetic nervous system might exacerbate these processes and cause MTrP formation. The sympathetic nervous system controls extrafusal and intrafusal muscle fibers through collateral branches (Selkowitz, 1992; Bombardi et al., 2006), and enhances the release of acetylcholine from motor nerve terminals that is mediated by α- and β-adrenoceptors on motor nerve terminals (Gerwin et al., 2004).

By contrast, a reduction in sympathetic activity by MTrP compression might suppress MTrP processes and the associated pain. Compression at MTrPs in the upper trapezius muscle has been shown to ameliorate the perception of pain and reduce spontaneous electrical activity (SEA) in motor end-plates at MTrPs (Kostopoulos et al., 2008). SEA is thought to be induced by excessive acetylcholine release (Gerwin et al., 2004). Pain at MTrPs and SEA is also decreased by the injection of sympatholytic agents, such as α1-adrenergic antagonists (Hubbard and Berkoff, 1993; McNulty et al., 1994; Hong and Simons, 1998) and relaxation techniques to reduce sympathetic activity (Banks et al., 1998). These findings suggest that suppression of sympathetic activity by MTrP compression might reduce acetylcholine release and decrease muscle contraction.

Furthermore, reduced local blood flow has been observed in trapezius myalgia and was correlated with pain intensity (Larsson et al., 1990), which might be ascribed to vasoconstriction due to increased sympathetic activity. Compression at MTrPs can induce reactive hyperemia in the MTrP region (Simons et al., 1999). Taken together, these results suggest that compression at MTrPs induces pain relief through inhibition of sympathetic activity, which (1) might increase the peripheral blood flow and subsequent removal of noxious substances, and (2) might block the excessive release of acetylcholine. However, other alternative hypotheses for generation of MTrPs such as the neurogenic hypothesis (Srbely, 2010) and the neurophysiologic hypothesis (Partanen et al., 2010) have been also proposed. Further studies are required to fully understand the role of sympathetic activity in myofascial pain syndrome.

### Effects of MTrP compression on prefrontal hemodynamic responses

In the present study, the Oxy-Hb concentration was significantly decreased in the PFC including the DMPFC during compression at MTrPs compared with Non-MTrPs, while there was no significant difference in Deoxy-Hb between the MTrP and Non-MTrP groups. Thus, the present results did not show typical hemodynamic changes (i.e., the response patterns of Oxy-Hb were not opposite to those of Deoxy-Hb concentration in the present study). However, changes in Deoxy-Hb were reported to be not consistent across individuals and across tasks (Hoshi et al., 2001; Toichi et al., 2004; Sato et al., 2005), while the Oxy-Hb concentration was stronger correlated with fMRI BOLD signals than Deoxy-Hb concentration (Strangman et al., 2002; Yamamoto and Kato, 2002). These findings suggest that Oxy-Hb concentration may be the most consistent parameter for cortical activity (Okamoto et al., 2006).

Decreases in fMRI signals were associated with local decreases in neuronal activity (Shmuel et al., 2006). Concentration of inhibitory neurotransmitter GABA was associated with decreases in signal intensity in fMRI (Northoff et al., 2007; Muthukumaraswamy et al., 2009). In NIRS studies, a decrease in Oxy-Hb concentration might correspond to inhibition of brain activity (Seitz and Roland, 1992; Shmuel et al., 2002; Stefanovic et al., 2004). Thus, the decrease in Oxy-Hb suggests that prefrontal activity was suppressed by compression at MTrPs. However, it is noted that compression at Non-MTrPs increased the Oxy-Hb concentration in the prefrontal cortex. The opposite hemodynamic responses in MTrP and Non-MTrP compression cannot be ascribed to differences in the psychophysical characteristics of compression between MTrPs and Non-MTrPs; there were no significant differences in compression intensity, pain intensity scores, and comfort/discomfort scores between MTrP and Non-MTrP compression. Further studies are required to determine the physiological factors contributing to differences in prefrontal hemodynamic responses between compression at MTrPs and Non-MTrPs.

Furthermore, changes in Oxy-Hb concentration in the DMPFC were significantly correlated with changes in HRV parameters (HF%, LF%, LF/HF; lnHF, lnLF, lnLF/HF) during compression (Figure 7, Supplementary Figure 2). A similar correlation has been reported for thermotherapy on the neck region (Yasui et al., 2010). Previous neuroanatomical and non-invasive imaging studies have reported that the PFC including the DMPFC has direct connections with the hypothalamus and brainstem, which are involved in autonomic and behavioral responses to pain (Ongür et al., 1998; Hadjipavlou et al., 2006). Increased activity in the DMPFC during the experience of pain has been correlated with decreased skin blood flow and increased skin conductance responses, suggesting increased sympathetic activity (Seifert et al., 2013). Furthermore, EEG gamma-band oscillation in the DMPFC and autonomic functions coherently changed in response to mental stress, and an increase in gamma-band oscillation went ahead of the autonomic fluctuation (Umeno et al., 2003). Chronic pain states such as chronic low back pain and sympathetically mediated chronic pain were found to be associated with hyperactivity in the mPFC including the DMPFC (Apkarian et al., 2001; Baliki et al., 2008). On the other hand, the dorsolateral PFC is implicated in inhibition of pain perception (Lorenz et al., 2003; Brighina et al., 2004), and gray matter atrophy in this brain region was reported in patients with chronic neck pain with MTrP and chronic low back pain (Fritz et al., 2016; Niddam et al., 2017). These findings suggest that unbalanced activity within the PFC might be associated with chronic pain. Thus, the available findings suggest that hyperactivity in the mPFC including the DMPFC might be involved in altered autonomic activity in the chronic pain state, and that compression at MTrPs might suppress sympathetic activity via the mPFC.

### Possible physiological mechanisms for the effects of MTrP compression

Different cerebral hemodynamic and autonomic nervous system responses were observed for compression at MTrPs and Non-MTrPs in the present study. The spatial locations of 70% of MTrPs have been reported to overlap with the locations of traditional acupuncture points (Melzack et al., 1997). Needling stimulation at both acupuncture points and MTrPs has been shown to significantly induce a stronger specific sensation known as “deqi” compared with needling stimulation at Non-MTrPs and non-acupuncture points (Roth et al., 1997; Takamoto et al., 2010). Deqi sensation is a composite of unique sensations described as aching, soreness, pressure, heaviness, fullness, warmth, cooling, numbness, tingling, and dull pain (Kong et al., 2007). Deqi sensations have also been induced by pressure stimulation at acupuncture points (Yip and Tse, 2004, 2006; Li et al., 2007). Furthermore, sustained pressure stimulation at MTrPs has also elicited deqi-like sensations including aching and dull pain in the distal or proximal areas from the point of stimulation (Simons, 1995; Clark, 2008; Delany, 2014). Thus, stimulation at both MTrPs and acupoints induces similar deqi sensations. MTrPs and acupuncture points are considered to be locations where the sensitization of polymodal-type receptors occurs (Kawakita and Itoh, 2002). Polymodal-type receptors are present on afferent fibers with low conduction velocity (A-delta and C-fibers) (Almeida et al., 2004), which might be involved in deqi sensations evoked by needling stimulation at acupuncture points (Lu, 1983; Wang et al., 1985). The physiological effects induced by compression at MTrPs in the present study might be attributed to deqi sensations.

Needling at acupuncture points that induced deqi sensations has been shown to increase local blood flow, whereas the stimulation of non-acupuncture points changes the local blood flow only slightly (Kuo et al., 2004). Furthermore, the number of episodes of deqi sensations induced by needling at acupuncture points was correlated with a decrease in sympathetic activity and an increase in parasympathetic activity (Sakai et al., 2007), and a significant negative correlation was observed between the intensity of the specific acupuncture sensations and heart rate responses (Beissner et al., 2012). A neuroimaging study reported that decreased activity in the prefrontal cortex was associated with both increased intensity of deqi sensation and heart rate responses during needling at acupuncture points (Beissner et al., 2012). Furthermore, heart rate responses were correlated with fMRI BOLD signals in the mPFC during needling at acupuncture points, and a greater decrease in mPFC activity was associated with a greater decrease in heart rates (Napadow et al., 2013). A previous NIRS study also showed that needling at MTrPs, which induced deqi sensations, decreased Oxy-Hb concentrations in the DMPFC and supplementary motor cortex (Takamoto et al., 2010). Taken together, different therapeutic methods employing either compression or needling at MTrPs and/or acupuncture points, might provide pain relief through common afferent nerve fibers that induce deqi sensations. Further studies are required to investigate the physiological mechanisms that induce cerebral hemodynamic and autonomic nervous system responses by compression at MTrPs.

## Conclusions

We conducted a pilot study to investigate the effects of compression at MTrPs in the neck region of patients with chronic neck pain on subjective pain perception, prefrontal hemodynamic activity, and possible autonomic activity using NIRS and HRV analyses. Compression at MTrPs significantly improved subjective pain scores compared with compression at Non-MTrPs. HRV parameters that are believed to reflect parasympathetic activity were significantly increased during compression at MTrPs compared with that induced by compression at Non-MTrP, and HRV parameters that are believed to reflect sympathetic activity were decreased. Furthermore, compression at MTrPs significantly decreased the Oxy-Hb concentration in the DMPFC compared with that induced by Non-MTrP compression. Changes in HRV parameters that are believed to reflect sympathetic activity were positively correlated with changes in Oxy-Hb concentrations in the DMPFC, and were positively correlated with changes in subjective pain scores during ischemic compression. The present results, along with those of previous studies, suggest that the effects of compression at MTrPs might be mediated through inhibitory effects on DMPFC activity, which might be beneficial for treating chronic pain in which hyperactivity of the sympathetic nervous system is involved.

### Limitations

First of all, we analyzed non-invasively HRV, but not by direct microneurography of sympathetic and parasympathetic nervous activity. This non-invasive method is widely used in clinical and experimental researches (Task Force of the European Society of Cardiology and the North American Society of Pacing and Electrophysiology, 1996; Lombardi and Stein, 2011), and normalized HRV parameters (HF%, LH%, LH/HF) were effective to evaluate muscle sympathetic nervous activity (Pagani et al., 1997). However, recent reviews argue that meaning of these HRV indices is more complex in that both LF and HF components may be modulated not only by sympathetic and parasympathetic neural activity but also by other non-autonomic nervous factors (Lombardi and Stein, 2011; Billman, 2013; Heathers, 2014; Gasior et al., 2016), which should be taken into account.

Second, it is noted that Oxy-Hb signals includes both cerebral (brain) and extra cerebral (scalp, skull, cerebrospinal fluid) components (Fukui et al., 2003). Our previous study using a multi-distance NIRS probe arrangement indicated that mean contribution ratio of the brain to whole hemodynamic signals at 3 cm of probe distance was 66.6% in the DMPFC (Ishikuro et al., 2014). Further studies were required to determine contribution ratio of the brain during compression at MTrPs to dissociate extra cerebral and cerebral components of the hemodynamic responses.

Finally, some of the correlations among autonomic nervous activity, neck pain and PFC hemodynamic activity were not high with a substantial variance in the data in the present study. Furthermore, causal relationships among these parameters remain unknown. Further studies with larger sample size, in which inhibition of the DMPFC using transcranial direct current stimulation (tDCS) should be also tested, are required.

## Author contributions

Designing work: HNishijo and KT; Data acquisition: YM and KT; Data analysis and interpretation: All of the authors.

### Conflict of interest statement

The authors declare that the research was conducted in the absence of any commercial or financial relationships that could be construed as a potential conflict of interest.
