# Supplementary material for: Compression at Myofascial Trigger Point on Chronic Neck Pain Provides Pain Relief through the Prefrontal Cortex and Autonomic Nervous System: A Pilot Study
Source: Front Neurosci. 2017 Apr 11;11:186. doi: 10.3389/fnins.2017.00186 (PMC5386976; doi:10.3389/fnins.2017.00186)
Supplement: Supplementary file 1 [file Presentation1.PDF]

Supplementary Material

Compression at myofascial trigger point on chronic neck pain provides immediate pain relief through the prefrontal cortex and autonomic nervous system: A pilot study

Yoshiki Morikawa, Kouich Takamoto, Hiroshi Nishimaru, Toru Taguchi, Susumu Urakawa, Shigekazu Sakai, Taketoshi Ono, Hisao Nishijo*

*** Correspondence: Dr. Hisao Nishijo, nishijo@med.u-toyama.ac.jp**

1. Supplementary figures

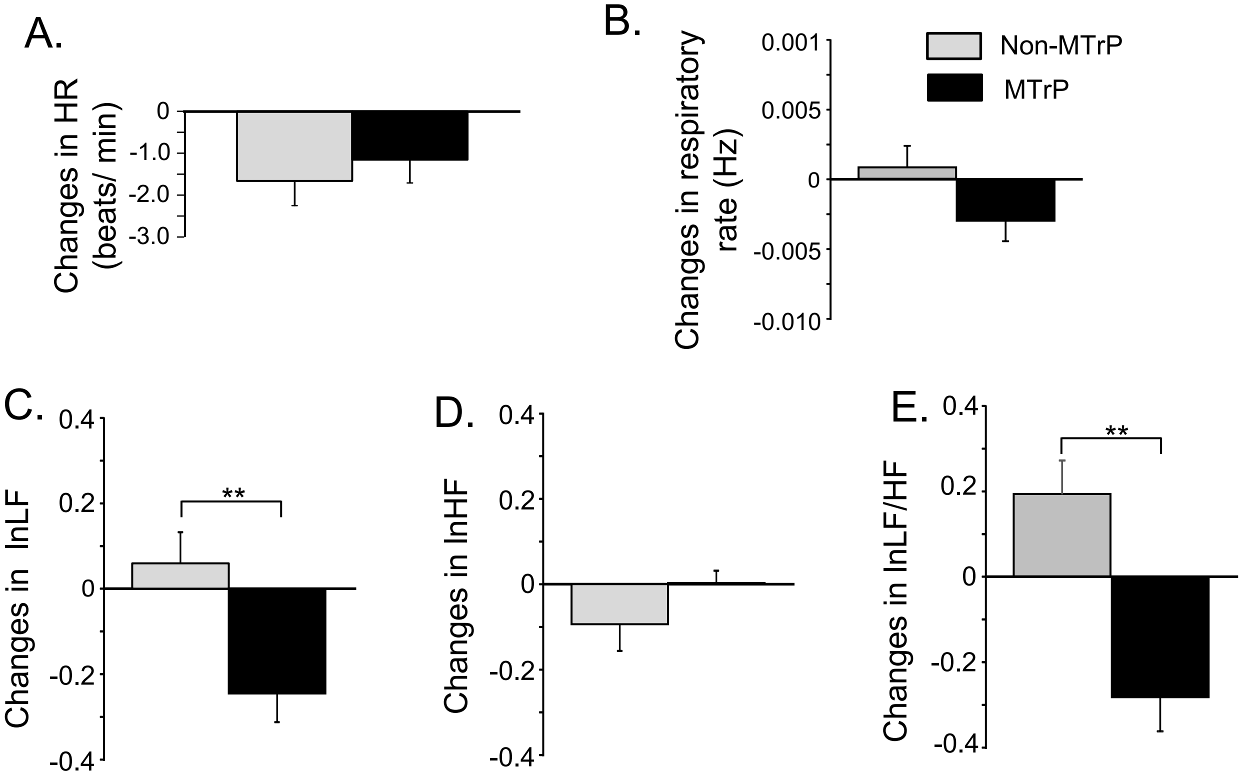

Supplementary Fig. 1. Comparison of changes in heart rates (HR) and respiratory rates (A, B) and changes in HRV parameters normalized by natural logarithm transformation (C-E) during compression between the MTrP and Non-MTrP groups.

(A) There was no significant difference in changes in heart rates during compression between the MTrPs and Non-MTrPs.

(B) There was no significant difference in changes in respiratory rates during compression between the MTrPs and Non-MTrPs.

(C) Changes in logarithmically transformed LF component (lnLF) were significantly decreased during compression at MTrPs compared with Non-MTrPs.

(D) There was no significant difference in changes in logarithmically transformed HF component (lnHF) during compression between the MTrPs and Non-MTrPs.

(E) Changes in logarithmically transformed LF/HF ratio (lnLF/HF) were significantly decreased during compression at MTrPs compared with Non-MTrPs. Error bars indicate SE; ** $P < 0.01$.

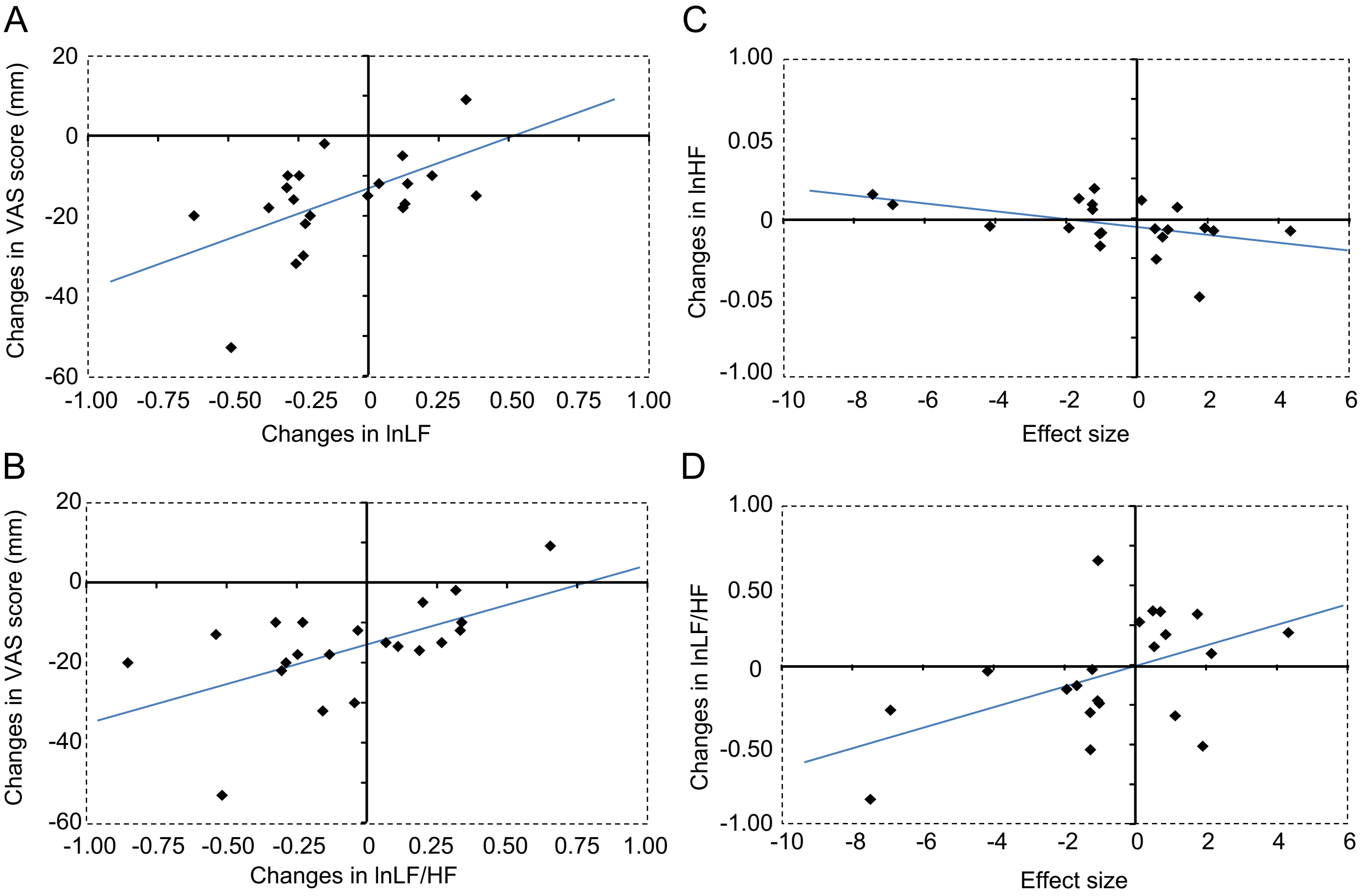

Supplementary Fig. 2. Correlations between changes in possible autonomic activity normalized by natural logarithm transformation (lnLF, lnLF/HF) and changes in subjective pain scores (A, B), and between changes in possible autonomic activity normalized by natural logarithm transformation and changes in hemodynamic responses in the DMPFC (C, D).

(A) A positive correlation was observed between changes in lnLF and changes in subjective pain scores.

(B) A positive correlation was observed between changes in lnLF/HF and changes in subjective pain scores. VAS, visual analog scale.

(C) A negative correlation was observed between changes in lnHF and changes in the hemodynamic response in the DMPFC.

(D) A positive correlation was observed between changes in lnLF/HF and changes in the hemodynamic response in the DMPFC.
